# Supplementary material for: Molecular weaving via surface-templated epitaxy of crystalline coordination networks
Source: Nat Commun. 2017 Feb 15;8:14442. doi: 10.1038/ncomms14442 (PMC5316853; doi:10.1038/ncomms14442)
Supplement: Supplementary Information — Supplementary Figures and Supplementary Methods [file ncomms14442-s1.pdf]

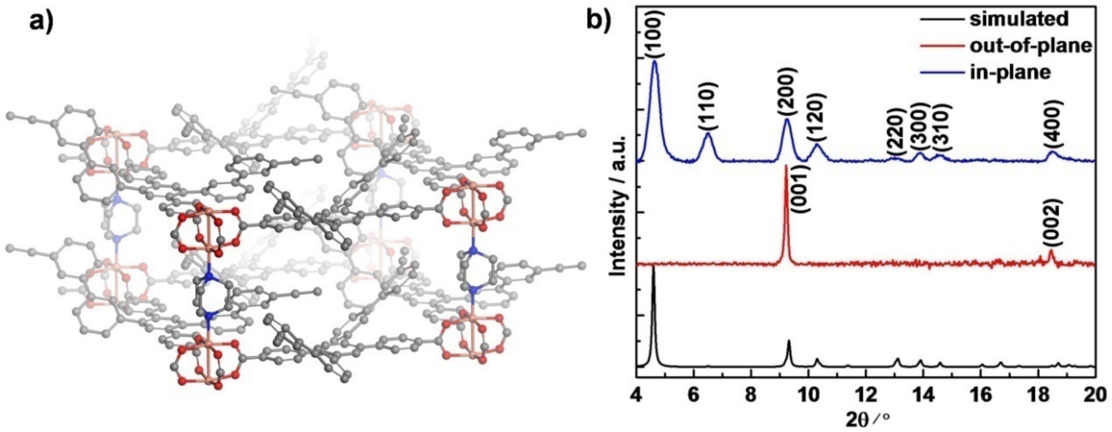

Supplementary Figure 1: Structure of Cu_2(BAB-TPDC)$_2$(Dabco) SURMOFs. a) Structure of Cu_2(BAB-TPDC)$_2$(Dabco). b) XRD patterns of Cu_2(BAB-TPDC)$_2$(Dabco) SURMOFs.

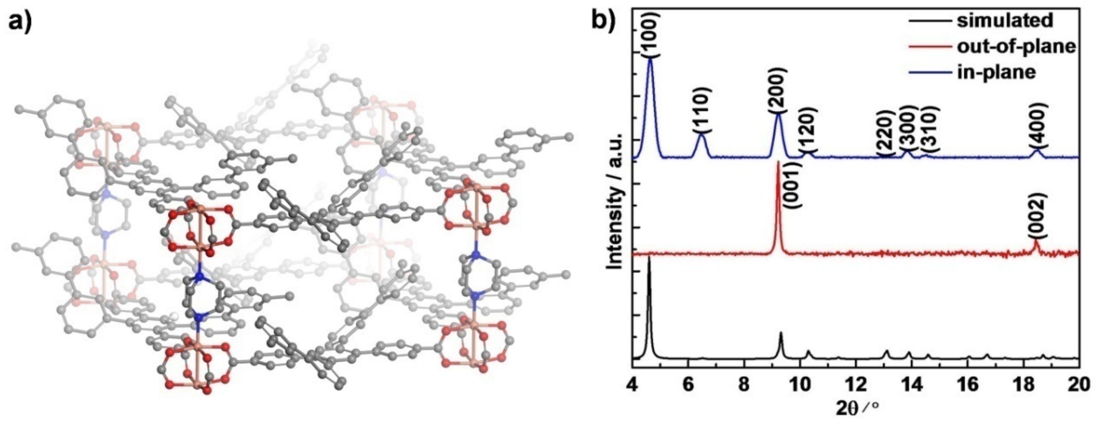

Supplementary Figure 2: Structure of $Cu_2(BAB\text{-}TPDC)_2(Dabco)$ SURMOFs. a) Structure of $Cu_2(BAB\text{-}TPDC)_2(Dabco)$. b) XRD patterns of $Cu_2(BAB\text{-}TPDC)_2(Dabco)$ SURMOFs.

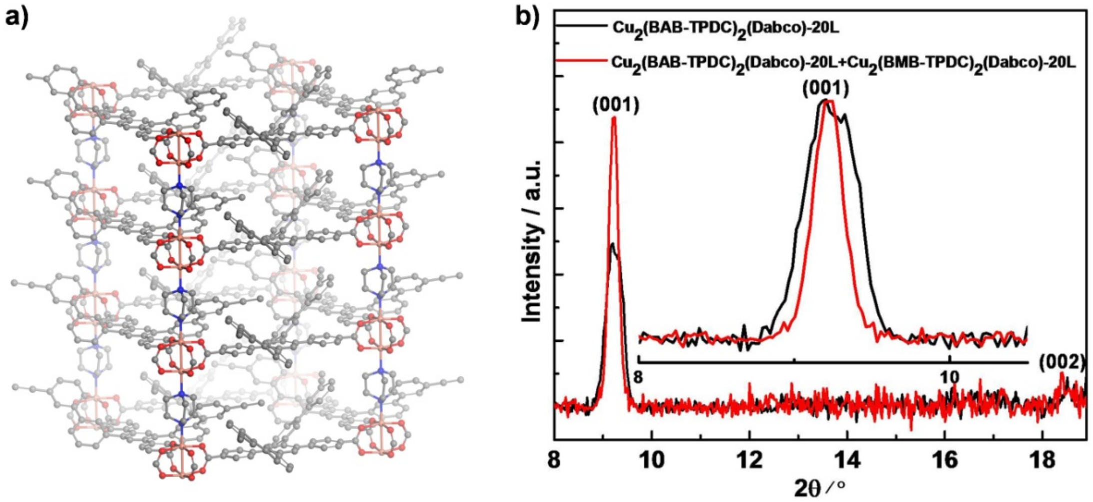

Supplementary Figure 3: Structure of Cu_2(BMB-TPDC)$_2$(Dabco) on Cu_2(BAB-TPDC)$_2$(Dabco) SURMOFs. a) Structure of Cu_2(BMB-TPDC)$_2$(Dabco) on Cu_2(BAB-TPDC)$_2$(Dabco) MOFs. b) XRD patterns of Cu_2(BAB-TPDC)$_2$(Dabco) SURMOFs and Cu_2(BMB-TPDC)$_2$(Dabco) SURMOFs on Cu_2(BAB-TPDC)$_2$(Dabco) SURMOFs. Inside is (001) peak with normalization, which demonstrate a increase of domain size of crystal after growing Cu_2(BMB-TPDC)$_2$(Dabco) SURMOFs on Cu_2(BAB-TPDC)$_2$(Dabco) SURMOFs according to Scherrer equation.

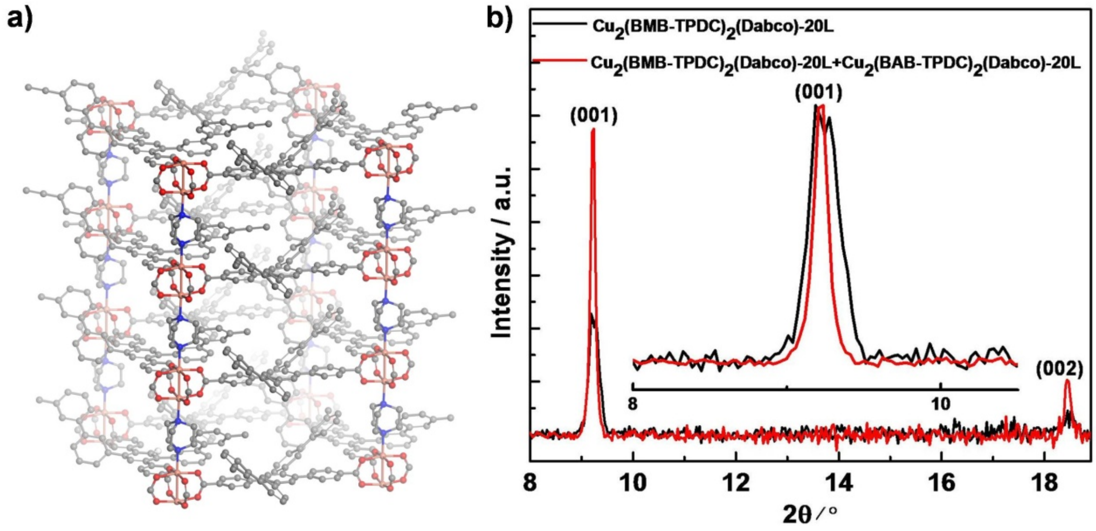

Supplementary Figure 4: Structure of Cu_2(BAB-TPDC)$_2$(Dabco). on Cu_2(BMB-TPDC)$_2$(Dabco) SURMOFs a) Structure of Cu_2(BAB-TPDC)$_2$(Dabco) on Cu_2(BMB-TPDC)$_2$(Dabco) MOFs. b) XRD patterns of Cu_2(BMB-TPDC)$_2$(Dabco) SURMOFs and Cu_2(BAB-TPDC)$_2$(Dabco) SURMOFs on Cu_2(BMB-TPDC)$_2$(Dabco) SURMOFs. Inside is (001) peak with normalization, which demonstrate a increase of domain size of crystal after growing Cu_2(BAB-TPDC)$_2$(Dabco) SURMOFs on Cu_2(BMB-TPDC)$_2$(Dabco) SURMOFs according to Scherrer equation.

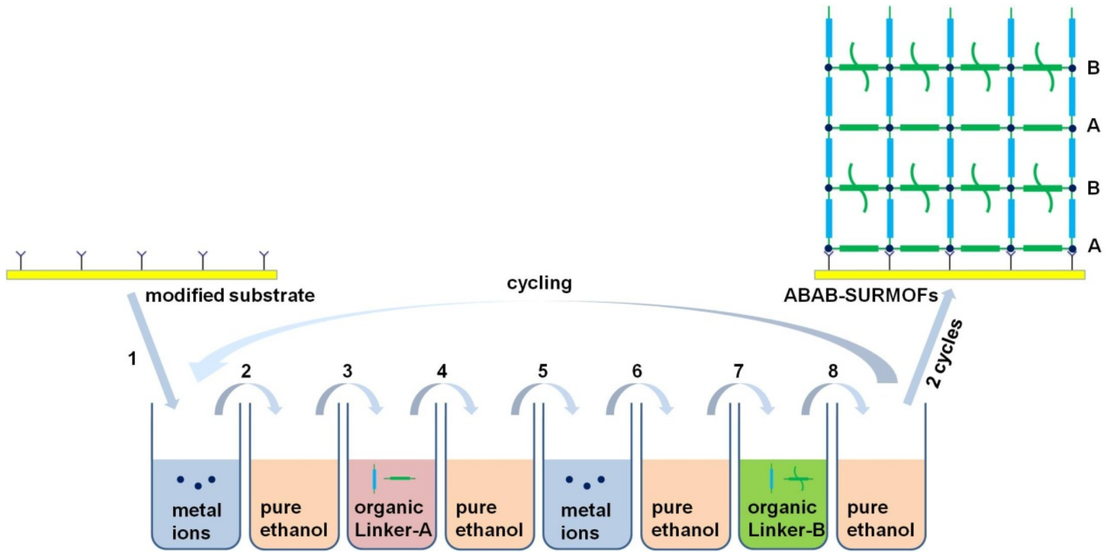

Supplementary Figure 5: ABAB-SURMOF preparation. Schematic illustration of the preparation of ABAB-SURMOFs

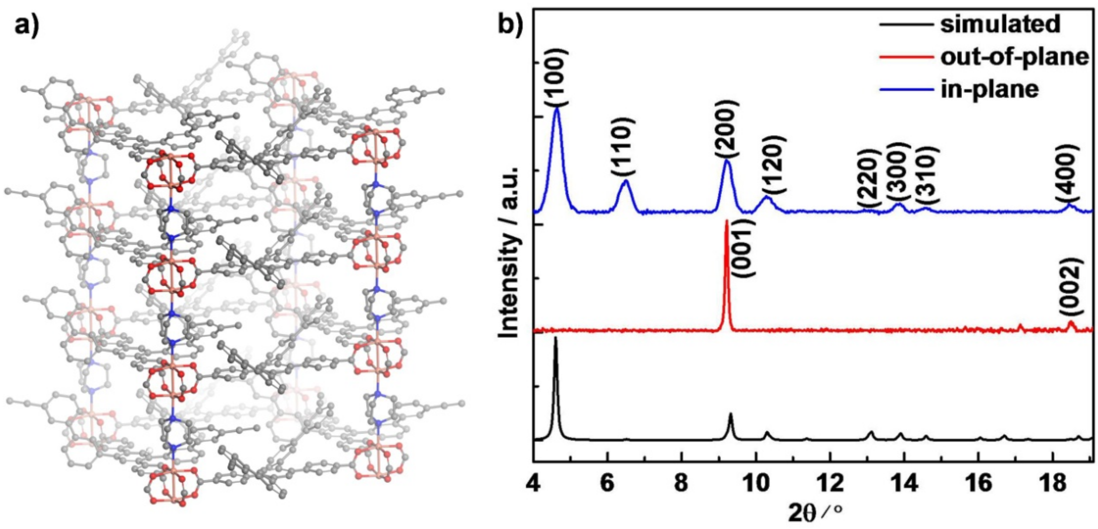

Supplementary Figure 6: Structure of ABAB-SURMOFs. **a**) Structure of ABAB-MOFs (A is $Cu_2(BAB\text{-}TPDC)_2(Dabco)$ and B is $Cu_2(BMB\text{-}TPDC)_2(Dabco)$ MOFs). b) XRD patterns of ABAB-SURMOFs.

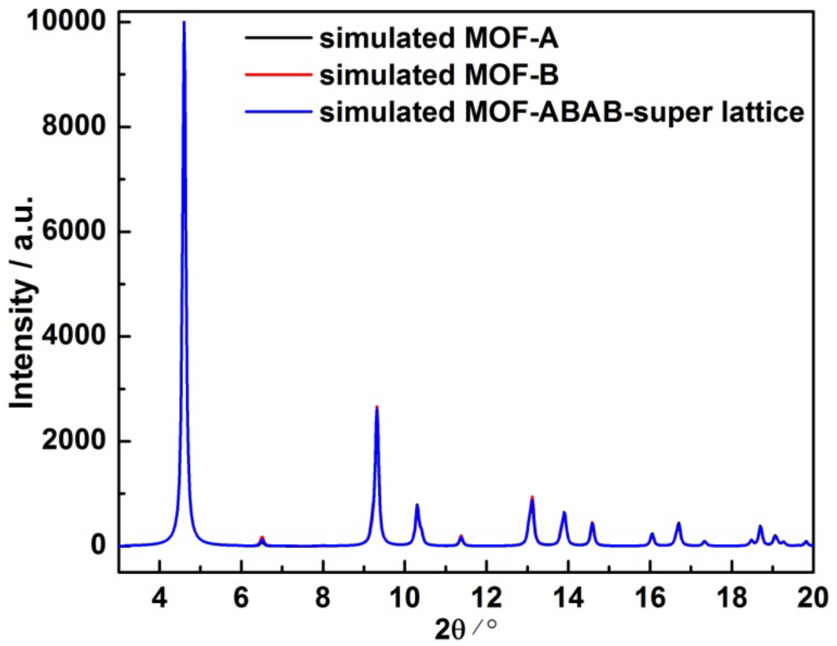

Supplementary Figure 7: Simulated XRD patterns. Comparison of the simulated XRD patterns of MOF-A, MOF-B and MOF-AB, which demonstrate the presence of only small differences of relative peak intensities.

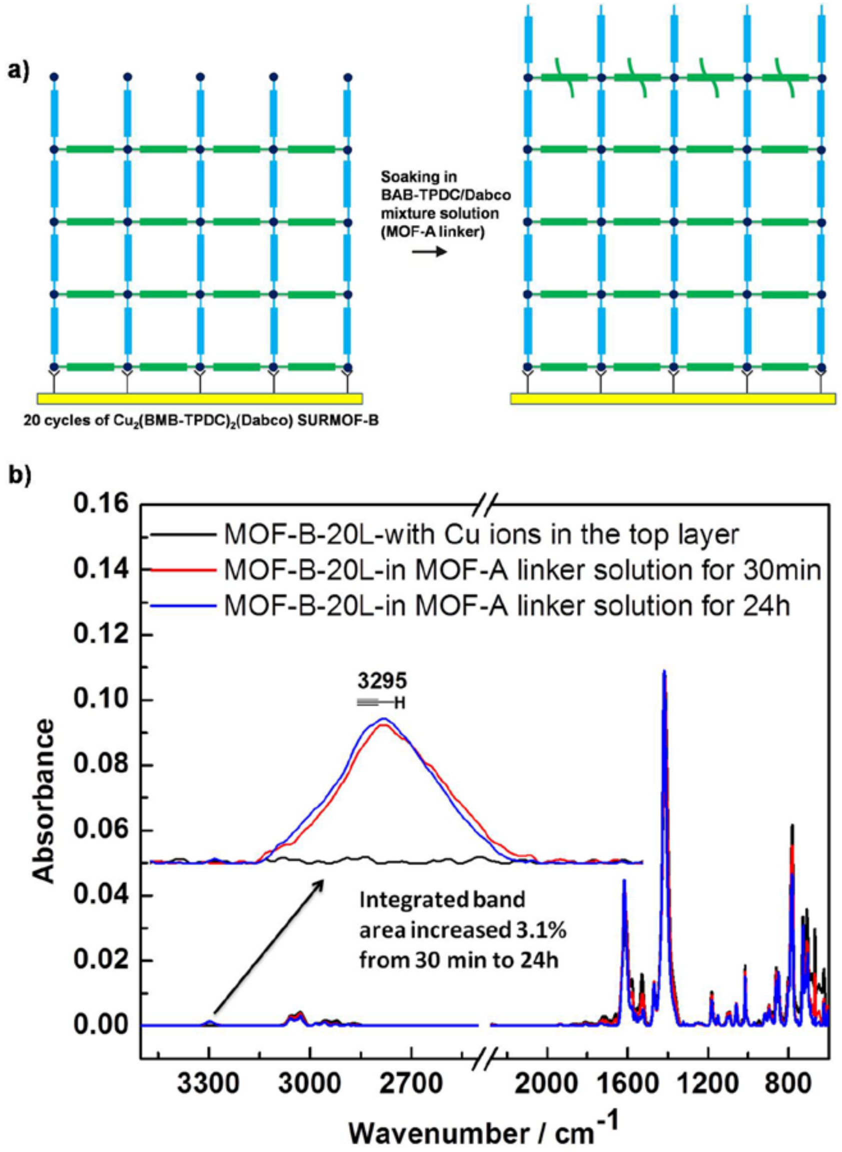

Supplementary Figure 8: Linker exchange of linker-B in MOF-B by linker-A. a) Schematic illustration of the soaking of 20 cycles of Cu_2(BMB-TPDC)$_2$(Dabco) SURMOF-B in MOF-A linker (BAB-TPDC/Dabco mixture: 0.05/0.05 mM) ethanol solution. b) IRRA spectra of 20 cycles of Cu_2(BMB-TPDC)$_2$(Dabco) SURMOF-B before and after soaking in MOF-A linker (BAB-TPDC/Dabco mixture: 0.05/0.05 mM) ethanol solution at 60 °C for 30 min and 24 h.

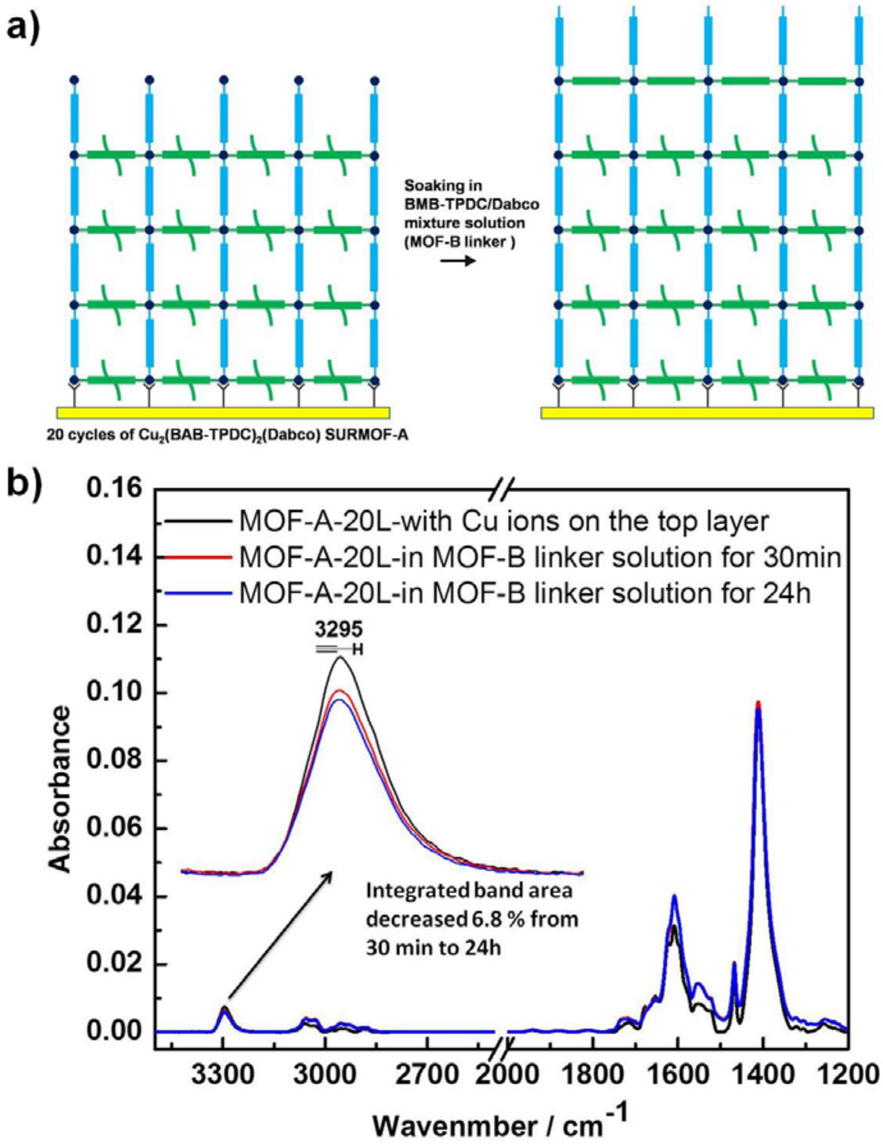

Supplementary Figure 9: Linker exchange of linker-B in MOF-A by linker-B. a) Schematic illustration of the soaking of 20 cycles of Cu_2(BAB-TPDC)$_2$(Dabco) SURMOF-A in MOF-B linker (BMB-TPDC/Dabco mixture: 0.05/0.05 mM) ethanol solution. b) IRRA spectra of 20 cycles of Cu_2(BAB-TPDC)$_2$(Dabco) SURMOF-B before and after soaking in MOF-B linker (BMB-TPDC/Dabco mixture: 0.05/0.05 mM) ethanol solution at 60 °C for 30 min and 24 h.

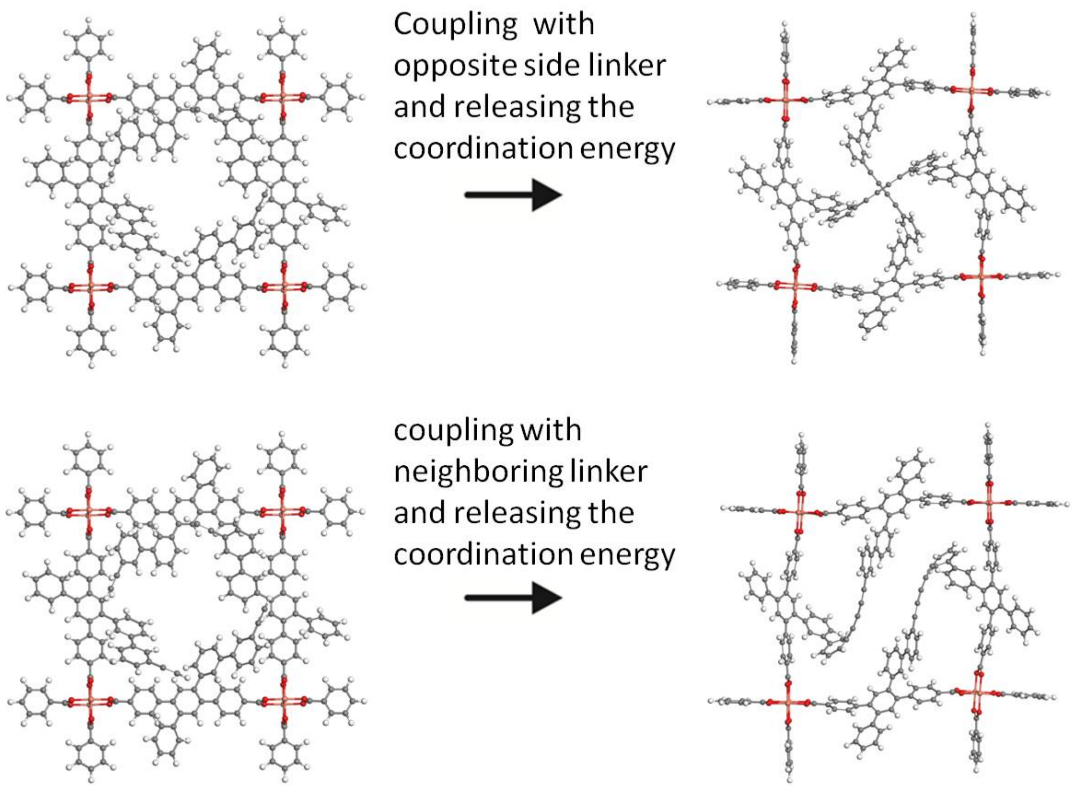

Supplementary Figure 10: Structure simulation. Simulated structure before and after cross-linking and releasing the coordination energy

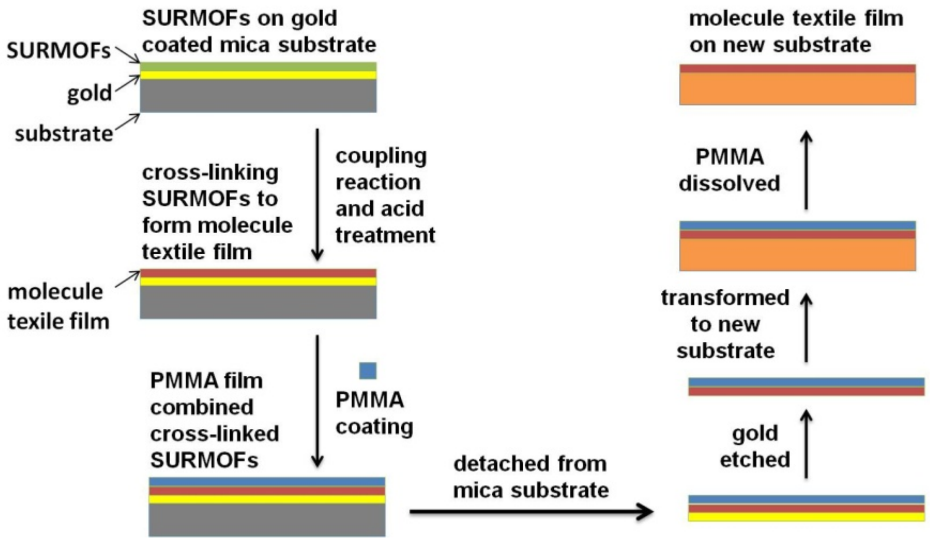

Supplementary Figure 11: Thin film transformation procedures. Transformation of molecule textiles to sacrificial substrate.

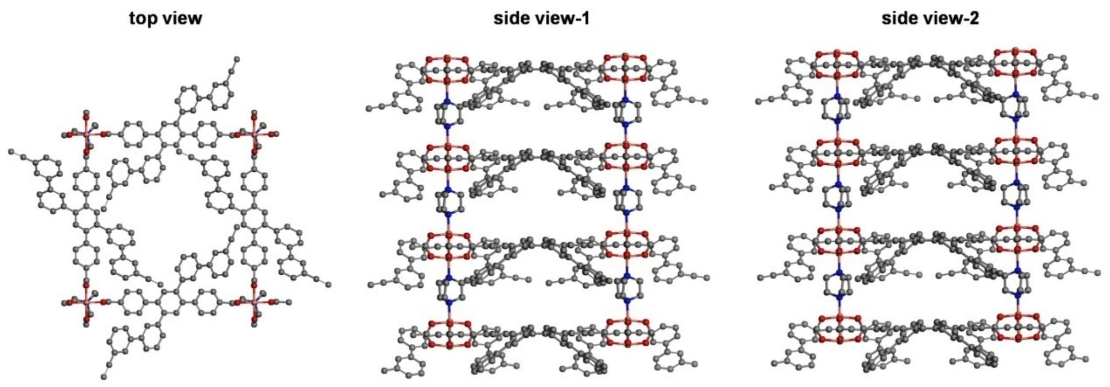

Supplementary Figure 12: Structure view of ABAB-SURMOFs. The top and side structure view of the ABAB-SURMOFs (A is Cu_2(BAB-TPDC)$_2$(Dabco) and B is Cu_2(BMB-TPDC)$_2$(Dabco) MOFs).

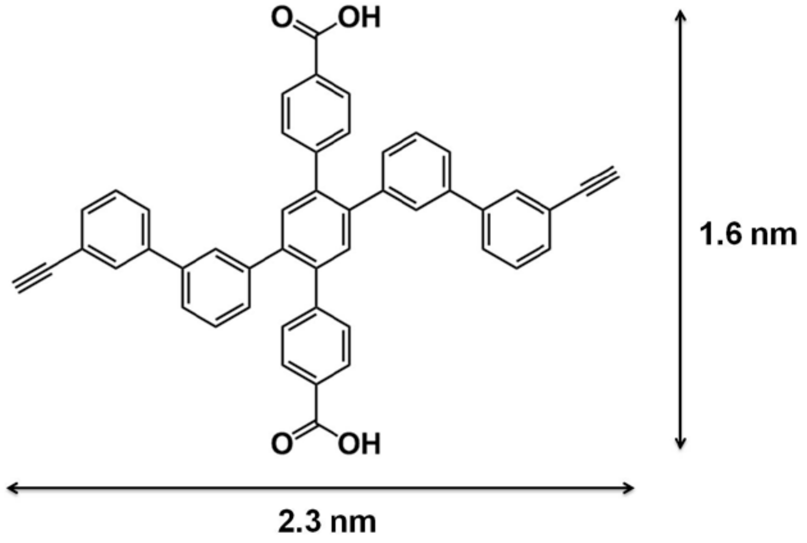

Supplementary Figure 13: The size of the organic linker monemor.

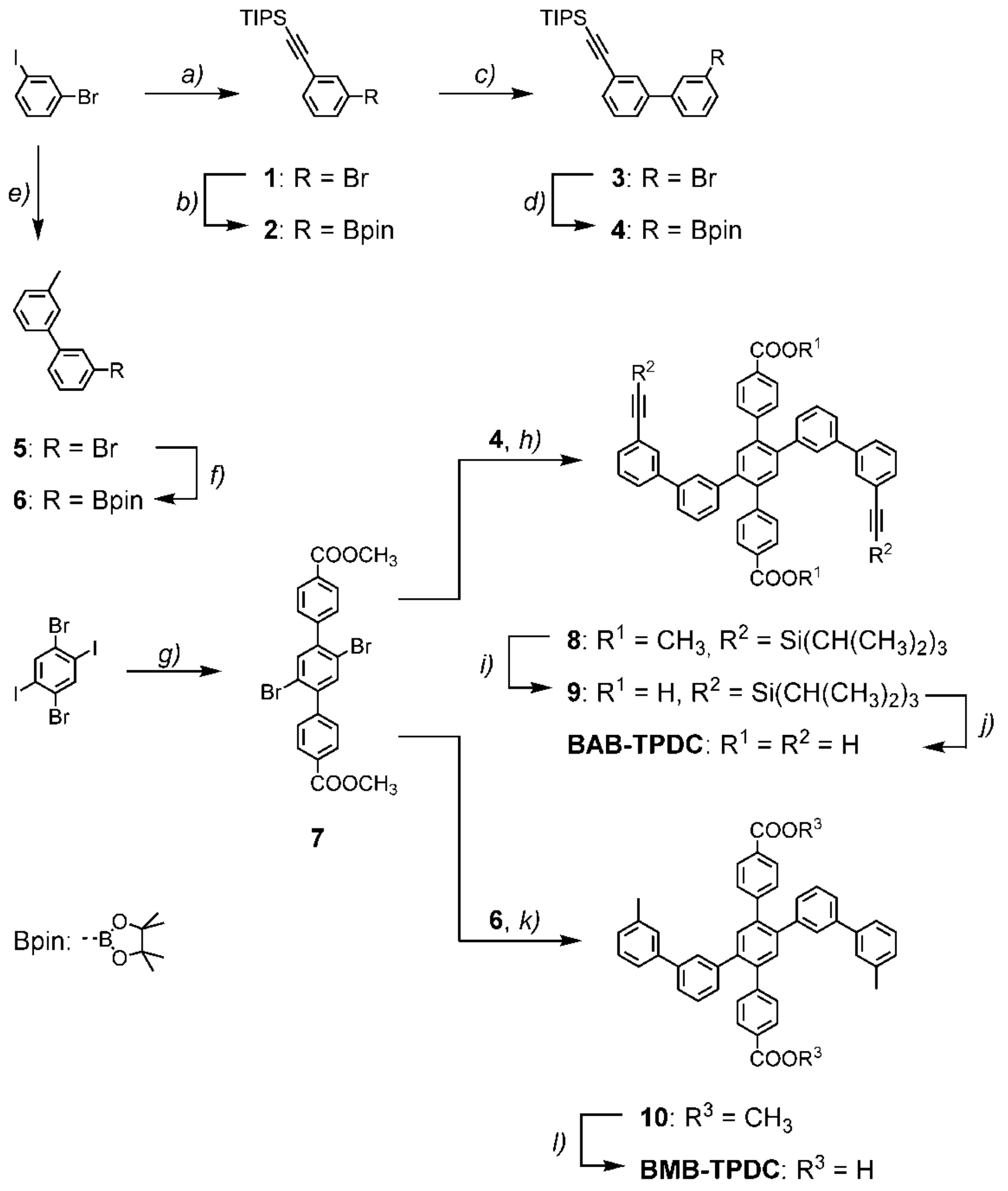

Supplementary Figure 14: Synthesis of BAB-TPDC and BMB-TPDC. Reagents and conditions: *a)* (trisisopropylsilyl)acetylene, $Pd(PPh_3)_4$, CuI, TEA, THF, reflux for 16h, 95%; *b)* bis(pinacolato)diboron, $Pd(dppf)Cl_2$ CH_2Cl_2, KOAc, dioxane, reflux for 16h, 94%; *c)* 1-bromo-3-iodobenzen, $Pd(PPh_3)_4$, K_2CO_3, DME/H_2O, 60°C, 24h, 74%; *d)* bis(pinacolato)diboron, $Pd(dppf)Cl_2$ CH_2Cl_2, KOAc, dioxane, 80°C, 16h, 82%; *e) m*-tolylboronic acid, $Pd(PPh_3)_2Cl_2$, K_2CO_3, DME/H_2O, 65°C, 16h, 86%; *f)* bis(pinacolato)diboron, $Pd(dppf)Cl_2{\cdot}CH_2Cl_2$, KOAc, dioxane, 90°C, 16h, 98%; *g)* 4-(methoxycarbonyl)-phenylboronic acid, $Pd(PPh_3)_2Cl_2$, K_2CO_3, DME/H_2O, 65°C, 24h, 38%; *h)* $Pd(PPh_3)_2Cl_2$, K_2CO_3, toluene/H_2O, 100°C, 24 h, 58%; *i)* NaOH, THF/MeOH, rt, 16h, 84%; *j)* TBAF, THF, rt, 3 h, 90%; *k)* $Pd(PPh_3)_2Cl_2$, K_2CO_3, DME/H_2O, reflux for 16h, 57%; *l)* NaOH, THF/H_2O, rt, 16h, 97%.

Supplementary method

Crystallographic date for BAB-TPDC. $C_{56}H_{46}O_6$ (*M* =814.93 g/mol): triclinic, space group $P\bar{1}$ (no. 2), a = 9.8382(8) Å, b = 12.5329(11) Å, c = 18.1034(15) Å, α = 83.594(7)°, β = 86.290(6)°, γ = 82.449(7)°, V = 2196.2(3) Å^3, Z = 2, T = 180.15 K, μ(MoKα) = 0.079 mm^{-1}, D_{calc} = 1.232 g/cm^3, 19725 reflections measured (2.27° ⩽ 2Θ ⩽ 51.44°), 8154 unique (R_{int} = 0.0254, R_{sigma} = 0.0248) which were used in all calculations. The final R_1 was 0.0710 (I > 2σ(I)) and wR_2 was 0.2301 (all data).

Synthesis of BAB-TPDC and BMB-TPDC. The syntheses of both SURMOF-building blocks is displayed in Supplementary Fig. 14. The assemblies of both building blocks, BAB-TPDC and BMB-TPDC, are mainly based on Pd catalyzed coupling chemistry, supplemented by conventional functional group transformation steps.

First, the biphenyl precursors 4 and 6 for the *meta*-quinquephenyl branches were assembled. Starting with commercially available 1-bromo-3-iodobenzene, the iodine was substituted with (trisisopropylsilyl)ethynyl using *Sonogashira* coupling conditions. The desired ethynylbenzene 1 was isolated in 95% yield after workup and column chromatography (CC) as colorless oil. Using *Suzuki-Myaura* conditions, 1 was treated with bis(pinacolato)diboron in presence of Pd(dppf)Cl_2 and KOAc in dry and degassed dioxane to provide the pinacolester 2 in 94% yield after workup and CC. The pinacolester 2 and 1-bromo-3-iodobenzene were coupled by *Suzuki* conditions to provide the biphenyl 3 in 74% yield as colorless oil after workup and CC. Again *Suzuki-Myaura* conditions were applied to transform the bromine of 3 into the boronic acid pinacolester 4, which was isolated in 82% as white solid after workup and CC. Also the synthesis of 6 starts with 1-bromo-3-iodobenzene, which was exposed together with *m*-tolylboronic acid to *Suzuki* coupling conditions to provide the biphenyl derivative 5 in 86% yield as colorless oil. Exposed to *Suzuki-Myaura* conditions, the biphenyl 5 was transformed to the boronic acid pinacolester 6, which was obtained in almost quantitative yield (98%) as yellowish oil.

With the precursors for the *meta*-quinquephenyl branches in hand, the focus moved on the assembly of the terphenyl backbone. Treating 1,4-dibromo-2,5-diiodobenzene with 4-(methoxycarbonyl)-phenylboronic acid in the presence of $Pd(PPh_3)_2Cl_2$ and K_2CO_3 in DME/H_2O mixture at 65°C gave the terphenyl structure 7, which was isolated as white solid after workup and CC in 38% yield. *Suzuki* coupling between 7 and 4 was performed in the presence of $Pd(PPh_3)_2Cl_2$ as catalyst and K_2CO_3 as base in a toluene/water mixture at 100°C and provided the fully protected target skeleton 8 as white solid in 58% yield after workup and CC. Subsequent hydrolization of both methyl esters of 8 with NaOH in THF/MeOH at room temperature provided the 4,4′′-dicarboxylic acid terphenyl derivative 9 as white solid in 84% yield. In a last step, 9 was treated with TBAF in wet THF at room temperature to provide the desired target compound BAB-TPDC, which was isolated by precipitation as whitish solid in 90% yield.

The ditopic linker BMB-TPDC was obtained by first treating the dibromoterphenyl 7 with the boronic acid pinacolester 6 in the presence of K_2CO_3 and catalytic amounts of $Pd(PPh_3)_2Cl_2$ in a DME/H_2O mixture to provide the terphenyl-quinquephenyl cruciform 10 as white solid in 57% yield after workup and CC. Subsequently, similar conditions as above described for 8 were applied to 10 to hydrolyze both esters, resulting in the desired ditopic linker BMB-TPDC as white solid in 97% yield.

General Remarks. All chemicals were used as received from the supplier, solvents were p.a. quality or dried and used without further purification. If necessary, the solvents were dried by standard literature procedures, THF with Na/benzophenone and Et_3N over CaH_2. The following instruments were used for the characterization of the synthesized compounds: ^{1}H-NMR and ^{13}C-NMR spectra were recorded on a Bruker NMR 500 Instrument, the *J* values are given in Hz. MALDI-TOF spectra was performed on a Waters Synapt time-of-flight mass spectrometer and EI-MS on a LKB-9000S. Melting points were measured with a Büchi Melting Point B-540 apparatus. TLC was carried out on Merck silica gel 60 F_{254} plates and column chromatography (CC) using Merck silica gel 60 (0.040-0.063 mm). Elemental analyses were performed using the ThermoQuest FlashEA 1112 N/Protein Analyzer.

((3-Bromophenyl)ethynyl)triisopropylsilane (1). The mixture of 1-bromo-3-iodobenzene (7.110 g, 25.1 mmol), $Pd(PPh_3)_2Cl_2$ (0.8809 g, 1.255 mmol), CuI (0.1 eq., 0.478 g, 2.51 mmol) and (trisisopropylsilyl)acetylene (1.1 eq., 5.0418 g, 27.6 mmol) in degassed THF/Et_3N (100/50 mL) was stirred at rt for 24 h. Then the solvents were removed by reduced pressure and the residue was purified by CC (silica gel, hexane) to get a desired product 1 in 95% yield (8.0145 g, colorless oil). ^{1}H NMR (500 MHz, $CDCl_3$, 25°C): δ = 1.10 (s, 3H, CH), 1.14 (s, 19H, CH_2), 7.17 (t, $^3J_{H,H}$ = 7.9 Hz, 1H), 7.40 (dt, $^3J_{H,H}$ = 7.8 Hz, $^4J_{H,H}$ = 1.1 Hz, 1H), 7.44 (dt, $^3J_{H,H}$ = 7.8 Hz, $^4J_{H,H}$ = 1.0 Hz, 1H), 7.63 (t, $^4J_{H,H}$ = 1.7 Hz, 1H) ppm. ^{13}C NMR (120 MHz, $CDCl_3$, 25°C): δ = 11.29, 18.63, 92.34, 105.28, 121.99, 125.49, 129.60, 130.56, 131.43, 134.69 ppm. $C_{17}H_{25}BrSi$ (337.38): calcd. C 60.52, H 7.47; found C 60.17, H 7.34.

Triisopropyl((3-(4,4,5,5-tetramethyl-1,3,2-dioxaborolan-2-yl)phenyl)ethynyl)silane (2). The mixture of 1 (4.0699 g, 12.1 mmol), bis(pinacolato)diboron (1.2 eq., 3.6761 g, 14.5 mmol), $Pd(dppf)Cl_2{\cdot}CH_2Cl_2$ (0.2 eq., 0.4942 g, 0.605 mmol) and KOAc (8 eq., 9.5 g, 96.8 mmol) in degassed dioxane (50 mL) was heated at 85°C for 16 h. Then the reaction mixture was poured into water and extracted with CH_2Cl_2 and washed with water. The solvents were removed by reduced pressure and the residue was purified by a short CC (silica gel, hexane) to afford 2 (4.3828 g) in 94% yield as colorless oil. ^{1}H NMR (500 MHz, CD_2Cl_2, 25°C): δ = 1.13 (s, 21H, CH, CH_3), 1.33 (s, 12H, CH_3), 7.32 (t, $^3J_{H,H}$ = 7.6 Hz, 1H), 7.54 (dt, $^3J_{H,H}$ = 7.8 Hz, $^4J_{H,H}$ = 1.5 Hz, 1H), 7.70 (d, $^3J_{H,H}$ = 7.4 Hz, 1H), 7.85 (s, 1H) ppm. ^{13}C NMR (120 MHz, $CDCl_3$, 25°C): δ = 11.68, 18.79, 25.00, 84.37, 90.74, 107.27, 123.34, 127.98, 134.75, 134.86, 138.31 ppm. $C_{23}H_{37}BO_2Si$ (384.44): calcd. C 71.86, H 9.70; found C 71.57, H 9.64; (EI): m/z (%): 384.2 (M^+, 3), 341.2 (100), 313.1 (25), 299.1 (41), 285.1 (36), 271.1 (60).

((3'-Bromo-[1,1'-biphenyl]-3-yl)ethynyl)triisopropylsilane (3). The mixture of 2 (4.3828 g, 11.4 mmol), 1-bromo-3-iodobenzene (1 eq., 2.9321 g, 10.36 mmol), $Pd(PPh_3)_2Cl_2$ (0.3636 g, 0.518 mmol) and K_2CO_3 (8 eq., 11.4548 g, 82.88 mmol) in degassed DME/H_2O (50 mL/10 mL) was heated at 60°C for 16 h. Then the reaction mixture was extracted with DCM and all solvents were removed by reduced pressure. The residue was purified by CC (silica gel, hexane) to get a desired product 3 as colorless oil in 74% yield (3.172 g). ^{1}H NMR (500 MHz, $CDCl_3$, 25°C): δ = 1.19 (s, 21H, CH, CH_3), 7.31 (t, $^3J_{H,H}$ = 7.8 Hz, 1H), 7.39 (t, $^3J_{H,H}$ = 7.8 Hz, 1H), 7.48-7.52 (m, 4H), 7.66 (st, $^4J_{H,H}$ = 1.5 Hz, 1H), 7.73 (st, $^4J_{H,H}$ = 1.8 Hz, 1H) ppm. ^{13}C NMR (120 MHz, $CDCl_3$, 25°C): δ = 11.30, 18.68, 91.09, 106.65, 122.91, 124.17, 125.77, 127.07, 128.76, 130.14, 130.28, 130.49, 130.57, 131.46, 139.81, 142.50 ppm. $C_{23}H_{29}BrSi$ (413.47): calcd. C 66.81, H 7.07; found C 66.51, H 7.04; (EI): m/z (%): 414.1 (M^+ + 2, 4), 412.1 (M^+, 4), 371.0 (100), 369.0 (100), 343.0 (22), 341.0 (23), 328.0 (40), 326.0 (40), 315.0 (54), 312.0 (51), 301 (80), 299 (74), 157.8 (55).

Triisopropyl((3'-(4,4,5,5-tetramethyl-1,3,2-dioxaborolan-2-yl)-[1,1'-biphenyl]-3-yl)ethynyl)-silane (4). The mixture of 3 (3.172 g, 7.67 mmol), bis(pinacolato)diboron (1.2 eq., 2.3377 g, 9.206 mmol),

$Pd(dppf)Cl_2 \cdot CH_2Cl_2$ (0.2 eq., 0.3132 g, 0.383 mmol) and KOAc (8 eq., 6.0219 g, 61.36 mmol) in degassed dioxane (150 mL) was heated at 80°C for 16 h. Then the reaction mixture was poured into water and extracted with CH_2Cl_2 and washed with water. The solvents were removed by reduced pressure and the residue was purified by a short CC (silica gel, CH_2Cl_2/hexane 1/1) to afford 4 (2.9051 g) in 82% yield as yellowish oil. ^1H NMR (500 MHz, CD_2Cl_2, 25°C): δ = 1.15 (s, 21H, CH, CH_3), 1.35 (s, 12H, CH_3), 7.39 (t, $^3J_{H,H}$ = 7.7 Hz, 1H), 7.42-7.48 (m, 2H), 7.58 (dt, $^3J_{H,H}$ = 7.3 Hz, $^4J_{H,H}$ = 1.3 Hz, 1H), 7.68 (dt, $^3J_{H,H}$ = 7.3 Hz, $^4J_{H,H}$ = 1.3 Hz, 1H), 7.72 (st, $^4J_{H,H}$ = 1.5 Hz, 1H), 7.76 (dt, $^3J_{H,H}$ = 7.3 Hz, $^4J_{H,H}$ = 1.1 Hz, 1H), 7.98 (s, 1H) ppm. ^{13}C NMR (120 MHz, CD_2Cl_2, 25°C): δ = 11.70, 18.80, 25.03, 84.29, 91.04, 107.31, 124.24, 127.67, 128.58, 129.04, 130.22, 130.79, 131.16, 133.58, 134.24, 139.94, 141.61 ppm. $C_{29}H_{41}BO_2$ (460.54): calcd. C 75.63, H 8.97; found C 75.39, H 8.88; (EI): m/z (%): 460.3 (M^+, 5), 417.2 (100), 389.2 (22), 347.2 (28), 375 (39), 261.1 (53), 247.1 (75).

3-Bromo-3'-methyl-1,1'-biphenyl (5). The mixture of 1-bromo-3-iodobenzen (13.56 mmol, 3.8358 g), m-tolylboronic acid (15.06 mmol, 2.0482 g), $Pd(PPh_3)_2Cl_2$ (0.753 mmol, 0.5285 g) and K_2CO_3 (6 eq, 90.36 mmol, 12.4886 g) in degassed DME/H_2O (20/15) was heated at 65°C for 16 h. The crude mixture was extracted with DCM and purified by chromatography column (silica gel, hexane) to get the product as colorless oil with 86% yield (2.871 g). ^1H NMR (500 MHz, $CDCl_3$, 25°C): δ = 2.45 (s, 3H), 7.21 (d, $^3J_{H,H}$ = 6.7 Hz, 1H), 7.31 (t, $^3J_{H,H}$ = 7.9 Hz, 1H), 7.34-7.41 (m, 3 H), 7.48 (d, $^3J_{H,H}$ = 8.0 Hz, 1H), 7.52 (d, $^3J_{H,H}$ = 7.7 Hz, 1H), 7.76 (t, $^4J_{H,H}$ = 1.6 Hz, 1H) ppm. ^{13}C NMR (120 MHz, $CDCl_3$, 25°C): δ = 21.5, 122.8, 124.2, 125.7, 127.8, 128.6, 128.8, 130.0, 138.5, 139.7, 143.5 ppm. $C_{13}H_{11}Br$ (247.14): calcd. C 63.18, H 4.49; found C 63.30, H 4.64. (EI): m/z (%): 248.0 (M^+ + 2, 95), 246.0 (M^+, 100), 167.1 (55), 165.1 (80), 152.1 (62).

4,4,5,5-Tetramethyl-2-(3'-methyl-[1,1'-biphenyl]-3-yl)-1,3,2-dioxaborolane (6). The mixture of 5 (2.6803 g, 10.84 mmol), bis(pinacolato)diboron (1.2 eq., 3.3048 g, 13 mmol), $Pd(dppf)Cl_2 \cdot CH_2Cl_2$ (0.05 eq., 0.4427 g, 0.542 mmol) and KOAc (4 eq., 4.2553 g, 43.36 mmol) in degassed dioxane (50 mL) was heated at 90°C for 16 h. Then the reaction mixture was poured into water and extracted with CH_2Cl_2 and washed with water. The solvents were removed by reduced pressure and the residue was purified by a short CC (silica gel, hexane and CH_2Cl_2/hexane 1/5) to afford 6 (3.1144 g) in 98% yield as yellowish oil. ^1H NMR (500 MHz, $CDCl_3$, 25°C): δ = 1.35 (s, 12 H), 2.42 (s, 3 H), 7.17 (d, $^3J_{H,H}$ = 7.5 Hz, 1H), 7.33 (t, $^3J_{H,H}$ = 7.5 Hz, 1H), 7.40-7.47 (m, 3 H), 7.69 (dt, $^3J_{H,H}$ = 7.8 Hz, $^4J_{H,H}$ = 1.3 Hz, 1H), 7.73 (dt, $^3J_{H,H}$ = 7.8 Hz, $^4J_{H,H}$ = 1.2 Hz, 1H), 8.0 (s, 1 H) ppm. $C_{19}H_{23}BO_2$ (294.20): calcd. C 77.57, H 7.88; found C 77.69, H 7.80. ^{13}C NMR (120 MHz, $CDCl_3$, 25°C): δ = 21.6, 25.1, 84.2, 124.5, 128.2, 128.3, 128.5, 129.0, 130.2, 133.6, 133.8, 138.8, 140.9, 141.3. (EI): m/z (%): 294.2 (M^+, 100), 279.1 (30), 208.1 (90), 194.1 (85).

Dimethyl 2',5'-dibromo-[1,1':4',1''-terphenyl]-4,4''-dicarboxylate (7). The mixture of 1,4-dibromo-2,5-diiodobenzene (2.3196 g, 4.75 mmol), 4-(methoxycarbonyl)-phenylboronic acid (2.2 eq, 1.8831 g, 10.5 mmol), $Pd(PPh_3)_2Cl_2$ (5% mmol, 0.2744 g) and K_2CO_3 (8 eq, 38 mmol, 5.252 g) in degassed DME/H_2O (50/10 mL) was heated at 65°C for 24 h. Then the reaction mixture was poured into water and extracted with DCM, than all solvents were removed by reduced pressure. The residue was purified by CC (silica gel, hexane/CH_2Cl_2 1:1) to get a desired product as colourless oil in 38% yield (0.8971g). Mp. 248-250°C. ^1H NMR (500 MHz, $CDCl_3$, 25°C): δ =1.57 (s, 6 H, OCH_3), 7.52 (d, $^3J_{H,H}$ = 8.3 Hz, 4H), 7.65 (s, 2 H), 8.13 (d, $^3J_{H,H}$ = 8.3 Hz, 4H) ppm. ^{13}C NMR (120 MHz, $CDCl_3$, 25°C): 52.3, 121.2, 129.4, 129.5, 129.9, 135.1, 142.5, 143.7, 166.7 ppm. $C_{22}H_{16}Br_2O_4$ (504.17): calcd. C 52.41, H 3.20; found C 52.27, H 3.19; (EI): m/z (%): 504.0 (M^+ + 3, 16), 501 (M^+, 10), 226.1 (100), 113.3 (51).

Dimethyl 2',5'-di (3'-triisopropylsilylethynyl- [1,1'-biphenyl]-3-yl)-[1,1':4',1''-terphenyl]-4,4''-dicarboxylate (8). The mixture of 4 (2.2 eq, 1.767 g, 3.84 mmol), 7 (0.8793 g, 1.744 mmol), $Pd(PPh_3)_2Cl_2$ (0.1224g, 0.174 mmol) and K_2CO_3 (8 eq., 1.9283 g, 13.95 mmol) in degassed toluene/H_2O (100/20 mL) was heated at 100°C for 24 h. Then the reaction mixture was poured into water and extracted with toluene, then all solvents were removed by reduced pressure. The residue was purified by CC (silica gel, hexane/CH_2Cl_2 1:1) to get the desired product 8 as whitish solid in 58 % yield (1.0309 g). Mp. 121-123°C. ^{1}H NMR (500 MHz, $CDCl_3$, 25°C): δ = 1.18 (s, 42H, CH, CH_3), 3.91 (s, 6H, OCH_3), 7.17 (d, $^3J_{H,H}$ = 7.7 Hz, 2H), 7.30-7.34 (m, 6H), 7.38 (d, $^3J_{H,H}$ = 8.3 Hz, 4H), 7.43-7.52 (m, 6H), 7.63 (d, $^3J_{H,H}$ = 8.3 Hz, 4H), 7.96 (d, $^3J_{H,H}$ = 8.3 Hz, 4H) ppm. ^{13}C NMR (120 MHz, $CDCl_3$, 25°C): δ = 11.44, 18.88, 52.21, 90.88, 107.02, 124.11, 125.99, 127.24, 128.67, 128.79, 129.19, 129.57, 130.06, 130.63, 131.17, 132.98, 139.52, 140.02, 140.53, 140.92, 145.59 ppm. $C_{68}H_{74}O_4Si_2$ (1011.51): calcd. C 80.75, H 7.37; found C 80.57, H 7.30; MS (MALDI-TOF): found: 1033.4286 m/z, calculated for $C_{68}H_{74}O_4Si_2Na$ 1033.5023.

2',5'-Di (3'-triisopropylsilylethynyl- [1,1'-biphenyl]-3-yl)- [1,1':4',1''-terphenyl]-4,4''-dicarboxylic acid (9). The mixture of 8 (1.097 g, 1.084 mmol) and NaOH (1.55 g) in CH_3OH/THF (100/50 mL) was stirred at rt for 16 h. Then the solvents were removed by reduced pressure and to the residue water was added and the solid was precipitated. It was filtered and dissolved in MeOH. Then during neutralization with sol. HCl the product precipitated as white solid (0.897 g, 84% yield). Mp. 284-286°C. ^{1}H NMR (500 MHz, DMSO-d_6, 25°C): δ = 1.10 (s, 42H, CH, CH_3), 7.20 (d, $^3J_{H,H}$ = 7.8 Hz, 2H), 7.32-7.45 (m, 10H), 7.51 (d, $^3J_{H,H}$ = 7.1 Hz, 2H), 7.55-7.61 (m, 6H), 7.64 (s, 2H), 7.3 (d, $^3J_{H,H}$ = 8.3 Hz, 4H), 12.92 (brs, 2H) ppm. ^{13}C NMR (120 MHz, DMSO-d_6, 25°C): δ = 10.72, 18.52, 90.19, 106.97, 122.98, 125.59, 127.45, 128.17, 128.88, 129.09, 129.18, 129.31, 129.56, 129.96, 130.90, 132.66, 138.99, 139.27, 140.32, 140.45, 144.81 ppm. $C_{66}H_{70}O_4Si_2$ (983.45): calcd. C 80.61, H 7.17; found C 80.47, H 7.14; MS (MALDI-TOF): found: 1005.3970 m/z, calculated for $C_{66}H_{70}O_4Si_2Na$ 1005.4710.

2',5'-Di(3'-ethynyl-[1,1'-biphenyl]-3-yl)-[1,1':4',1''-terphenyl]-4,4''-dicarboxylic acid (BAB-TPDC). To the solution of 9 (0.1353 g, 0.14 mmol) in wet THF (20 mL) TBAF (6 eq., 0.84 mmol, 0.265 g) was added and the reaction mixture was stirring for 3 h at room temperature. Then water was added to precipitate and the white solid was filtered. The crude product was dissolved in THF and the solution was neutralized with sol. HCl and water was added to precipitate the product, which was collected by filtration as whitish solid in 90 % yield. Mp. < 410°C. ^{1}H NMR (500 MHz, DMSO-d_6, 25°C): δ = 4.2 (s, 2 H, ≡CH), 7.23 (d, $^3J_{H,H}$ = 7.7 Hz, 2H), 7.36-7.46 (m, 10 H), 7.49-7.52 (m, 6H), 7.56 (d, $^3J_{H,H}$ = 7.8 Hz, 2H), 7.67 (s, 2H), 7.86 (d, $^3J_{H,H}$ = 8.2 Hz, 4H), 12.94 (bs, 2H) ppm. ^{13}C NMR (120 MHz, DMSO-d_6, 25°C): δ = 67.0, 81.0, 83.2, 122.4, 125.6, 127.4, 128.3, 129.0, 129.2, 129.3, 129.9, 130.1, 130.7, 132.6, 139.0, 139.1, 139.2, 140.3, 144.9, 167.1 ppm. $C_{48}H_{30}O_4$ (670.76): calcd. C 85.95, H 4.51; found C 85.71, H 4.47; MS (MALDI-TOF): found: 693.1372 m/z, calculated for $C_{48}H_{30}O_4Na$ 693.2042.

Dimethyl 2',5'-di(3'-methyl-[1,1'-biphenyl]-3-yl)-[1,1':4',1''-terphenyl]-4,4''-dicarboxylate (10). The mixture of 6 (5.26 mmol, 1.5475 g), 7 (2.1 mmol, 1.0608 g), $Pd(PPh_3)_2Cl_2$ (0.105 mmol, 0.0737 g) and K_2CO_3 (6 eq, 12.6 mmol, 1.7414 g) in degassed DME/H_2O (35/5 mL) was refluxed for 16 h. The crude mixture was extracted with DCM and purified by chromatography column (silica gel, hexane/DCM 1:1) to get the product as white solid in 57% yield (0.8184 g). M.p. 221-222°C. ^{1}H NMR (500 MHz, $CDCl_3$, 25°C): δ = 2.36 (s, 6H, CH_3), 3.91 (s, 6H, OCH_3), 7.08 (s, 2 H), 7.13 (d, $^3J_{H,H}$ = 7.4 Hz, 2H), 7.19-7.20 (m, 4 H), 7.28 (t, $^3J_{H,H}$ = 7.6 Hz, 2H), 7.33 (t, $^3J_{H,H}$ = 7.7 Hz, 2H), 7.38 (d, $^3J_{H,H}$ = 8.2 Hz, 2H), 7.42 (s, 2 H), 7.47 (d, $^3J_{H,H}$ = 7.7 Hz, 2 H), 7.65 (s, 2 H), 7.98 (d, $^3J_{H,H}$ = 8.2 Hz, 2H) ppm. ^{13}C NMR (120 MHz, $CDCl_3$, 25°C): δ

= 21.4, 52.1, 124.1, 125.9, 128.0, 128.1, 128.47, 128.52, 128.59, 128.62, 128.9, 130.0, 132.7, 138.3, 139.4, 139.8, 140.4, 140.7, 141.2, 145.8, 166.9 ppm. $C_{48}H_{38}O_4$ (678.83): calcd. C 84.93, H 5.64; found C 84.69, H 5.70. MS (MALDI-TOF): found: 701.2065 m/z, calculated for $C_{48}H_{38}O_4Na$ 701.2668.

2',5'-Di(3'-methyl-[1,1'-biphenyl]-3-yl)-[1,1':4',1''-terphenyl]-4,4''-dicarboxylic acid (BMB-TPDC). The mixture of 10 (0.4548 g, 0.67 mmol) and NaOH (1.2456 g) in THF/H_2O (30/10 mL) was stirred at rt for 16h. Then, sol. HCl was added and precipitate was formed, which was filtered off and washed with water to get the final product as a white solid (97% yield, 0.4236 g). M.p. 348-350°C. ^{1}H NMR (500 MHz, DMSO-d$_6$, 25°C): δ = 2.28 (s, 6H, CH_3), 6.99 (s, 2H), 7.11 (d, $^{3}J_{H,H}$ = 7 Hz, 2H), 7.23 (t, $^{3}J_{H,H}$ = 7.5 Hz, 4H), 7.30 (d, $^{3}J_{H,H}$ = 7.5 Hz, 2H), 7.35 (s, 2H), 7.39 (t, $^{3}J_{H,H}$ = 7.5 Hz, 2H), 7.42 (d, $^{3}J_{H,H}$ = 8.5 Hz, 2H), 7.50 (d, $^{3}J_{H,H}$ = 8 Hz, 2H), 7.65 (s, 2H), 7.89 (d, $^{3}J_{H,H}$ = 6.5 Hz, 4H), 12.9 (s, 2H, COOH) ppm. ^{13}C NMR (120 MHz, DMSO-d$_6$, 25°C): δ = 20.9, 123.7, 125.5, 127.4, 128.1, 128.4, 128.7, 129.0, 129.2, 129.3, 130.0, 132.4, 137.9, 139.1, 139.2, 139.91, 139.95, 140.0, 145.1, 167.1 ppm. $C_{46}H_{34}O_4$ (650.77): calcd. C 84.90, H 5.27; found C 85.10, H 5.40. MS (MALDI-TOF): found: 673.1683 m/z, calculated for $C_{46}H_{34}O_4Na$ 673.2355.
